# Tinnitus and Headache

**DOI:** 10.1155/2015/797416

**Published:** 2015-10-25

**Authors:** Berthold Langguth, Verena Hund, Volker Busch, Tim P. Jürgens, Jose-Miguel Lainez, Michael Landgrebe, Martin Schecklmann

**Affiliations:** ^1^Department of Psychiatry and Psychotherapy, University of Regensburg, 93049 Regensburg, Germany; ^2^Department of Systems Neuroscience, University Medical Center Hamburg-Eppendorf, 20246 Hamburg, Germany; ^3^Department of Neurology, Catholic University of Valencia, 46010 Valencia, Spain; ^4^Department of Psychiatry, Psychosomatics and Psychotherapy, Kbo-Lech-Mangfall-Klinik Agatharied, 83734 Hausham, Germany

## Abstract

*Background*. Tinnitus and headache are frequent disorders. Here, we aimed to investigate whether the occurrence of headache among tinnitus patients is purely coincidental or whether tinnitus and headache are pathophysiologically linked. We investigated a large sample of patients with tinnitus and headache to estimate prevalence rates of different headache forms, to determine the relationship between tinnitus laterality and headache laterality, and to explore the relationship between tinnitus and headache over time. *Method*. Patients who presented at a tertiary referral center because of tinnitus and reported comorbid headache were asked to complete validated questionnaires to determine the prevalence of migraine and tension-type headache and to assess tinnitus severity. In addition, several questions about the relationship between headache and tinnitus were asked. *Results*. Datasets of 193 patients with tinnitus and headache were analysed. 44.6% suffered from migraine, 13% from tension-type headache, and 5.7% from both. Headache laterality was significantly related to tinnitus laterality and in the majority of patients fluctuations in symptom severity of tinnitus and headache were interrelated. *Conclusion*. These findings suggest a significant relationship between tinnitus and headache laterality and symptom interaction over time and argue against a purely coincidental cooccurrence of tinnitus and headache. Both disorders may be linked by common pathophysiological mechanisms.

## 1. Introduction

Tinnitus is defined as the perception of sounds in the absence of a corresponding acoustic signal. It is a frequent disorder, which is reported by about 10% of the population [1]. While some patients can habituate or learn to ignore the phantom sound, others are severely impaired by their tinnitus. Previous research has shown that comorbidities such as hyperacusis [2], hearing loss [3], insomnia [4], depression [5, 6], pain syndromes [7], and headache [8, 9] play a major role in tinnitus-related impairment in quality of life [10]. Tinnitus-related impairment in quality of life can be measured by specific validated tinnitus questionnaires such as the “Tinnitus Questionnaire” [11] or the “Tinnitus Handicap Inventory” [12], but also by numeric rating scales [13, 14].

Like in headaches, idiopathic and secondary forms of tinnitus can be distinguished [15]. Several pathologies can cause both symptomatic headache and tinnitus, such as carotid artery dissections [16, 17], arteriovenous malformations, traumatic brain injury, space occupying intracranial lesions, and intracranial hypo- or hypertension [18]. Interestingly, many patients with idiopathic tinnitus report headache syndromes as well [18, 19]. Since both idiopathic headaches and idiopathic tinnitus are frequent disorders [20, 21], this could be purely coincidental. However, a large population-based epidemiological study in elderly people identified a history of migraine as clinical risk factor for the development of tinnitus and suggested an interrelation between tinnitus and headache syndromes [22].

Moreover, there is increasing evidence that some forms of idiopathic headaches and tinnitus share similar pathophysiological mechanisms. Both animal studies and human imaging studies found that tinnitus is related to abnormal activity in the central auditory pathways as a consequence of auditory deafferentation [23–25]. In addition to activity changes in central auditory pathways, alterations in a complex network of attention-, emotion-, and memory-related brain areas have been demonstrated [15, 26] resembling changes in a similar network of cortical areas in chronic pain [23–25, 27]. Moreover, it has been proposed that pain, headache, and tinnitus overlap in their pathophysiological mechanism by sharing specific alterations in thalamocortical activity [28–32]. These neurophysiological alterations which can be detected as specific changes of oscillatory activity by magneto- or electroencephalography have been described with the term thalamocortical dysrhythmia [29].

More recently, animal studies have demonstrated that trigeminal input interacts at the dorsal cochlear nucleus with the activity of central auditory pathways [33] and tinnitus perception, as assessed by behavioural tests [34]. Further support for an involvement of the trigeminal system in tinnitus pathophysiology derives from the clinical observation that many patients can modulate tinnitus activity by face and jaw movements [35, 36]. Moreover, an association between temporomandibular joint disorders and tinnitus [37, 38] is well established and successful treatment of temporomandibular joint disorders was shown to improve tinnitus [39]. Naturally, an abnormal function of the peripheral and central parts of the trigeminal system is a prerequisite for the formation of primary and secondary headaches, as has been shown in migraine [40] and trigeminal autonomic headaches [41]. Finally, recent studies identified shared pivotal clinical symptoms in Meniere's disease and vestibular migraine such as episodic hearing impairment, tinnitus, and vertigo [42, 43]. Thus, both the central pain processing network (also referred to as the “pain matrix”) and the trigeminal system represent a common link in the pathophysiology of idiopathic headache syndromes and tinnitus.

To further investigate a potential relationship between idiopathic headache and tinnitus, we asked patients who presented at the multidisciplinary Tinnitus Center at the University of Regensburg and who reported the existence of headaches in the Tinnitus Case History Questionnaire [44] to complete a headache questionnaire [45, 46] and to answer additional questions about the relationship between tinnitus and headache.

In detail, we aimed (1) to estimate prevalence rates of different headache forms among tinnitus patients, (2) to investigate whether there is a relationship between tinnitus laterality and headache laterality in patients with unilateral tinnitus and unilateral headache, and (3) to explore the relationship between tinnitus and headache over time.

## 2. Methods and Materials

### 2.1. Sample

The cross-sectional observational study was based on datasets of all patients aged between 18 and 90, who presented to the multidisciplinary Tinnitus Center of the University of Regensburg between 2003 and 2011 and whose data were included in the Tinnitus Research Initiative database [47]. All patients who reported the existence of headaches in the Tinnitus Case History Questionnaire (answer “yes” to the question, “Do you suffer from headaches?”) [44] were contacted by mail and asked to complete additional questionnaires. Informed consent was obtained before inclusion into the study. The study was approved by the ethics committee of the University of Regensburg (11-101-0286). All data were pseudonymised before further analysis.

### 2.2. Assessment of Headaches and Tinnitus Severity

In addition to the information available from the Tinnitus Research database [47], the actual tinnitus severity was quantified by the Tinnitus Questionnaire (TQ; Goebel 1994). According to the TQ score tinnitus severity can be classified as mild (0–30), moderate (31–46), severe (47–59), and extreme (60–84).

For classification of headaches, the diagnostic headache questionnaire of Fritsche et al. [45] was used, which was developed and validated to meet the diagnostic criteria for migraine and tension-type headache, 2nd version of the classification criteria of the International Headache Society (ICHD-2). The questionnaire enables differentiating migraine, tension-type headache, cluster headache, combination of migraine and tension-type headache, combination of tension-type and cluster headache, and nonclassifiable headache with very good test-retest reliability (0.95). Validation of the questionnaire in a tertiary headache clinic [48] and in a population-based sample [46] revealed that sensitivity and specificity of the questionnaire are sufficient to diagnose migraine and tension-type headache, but not trigeminal autonomic cephalgias.

Additional questions regarding headache frequency, headache medication, and the temporal relationship between tinnitus and headache were asked (Table 1; questions are provided in Supplementary Material available online at http://dx.doi.org/10.1155/2015/797416). Questions 29 and 30 of the Supplementary Material asked whether the onset of tinnitus influenced headache and vice versa. We combined these two questions into one variable whether beginning of the second symptom influenced the first symptom.

### 2.3. Statistical Analysis

The frequencies of the different headache forms and of the mutual interaction between tinnitus and headache in the sample were analysed descriptively. The relationship between tinnitus laterality and headache laterality was analysed by a Chi-square test of independence. The influence of a mutual interaction between tinnitus and headache on tinnitus severity was analysed by a one-factorial ANOVA. In case of significant results Fisher's least significant difference (LSD)* post hoc* tests were performed. A *p* value of <0.05 was regarded as statistically significant.

## 3. Results

**Figure 1:**
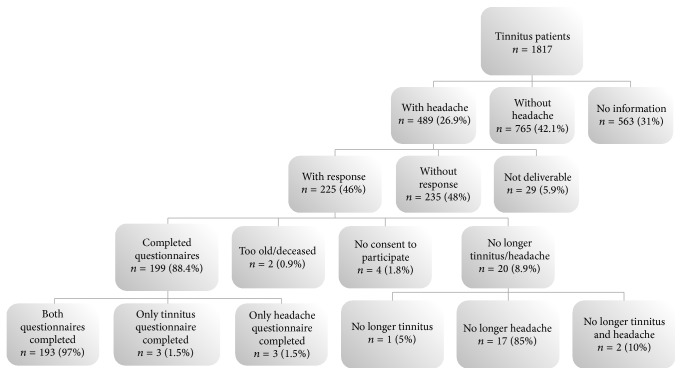
Data flow diagram of the questionnaire survey.

489 out of 1817 patients reported headaches in the Tinnitus Case History Questionnaire. All these 489 patients were contacted. 225 (46%) answered and 193 datasets were analyzed (for more information, see Figure 1).

The 193 participating patients (117 (60.6%) female, 52 ± 12 years) suffered for 97.3 ± 110.1 months from their tinnitus; their TQ score was 45.5 ± 18.3. 79 patients suffered from unilateral or predominantly unilateral tinnitus (51 left-sided, 28 right-sided); in 111 patients, the tinnitus was either on both sides or in the head (nonunilateral); three patients provided no information concerning tinnitus laterality.

According to the headache questionnaire, 86 (45%) patients suffered from migraine, 25 (13%) from tension-type headache, 8 (4%) from trigeminal autonomic headache, 11 (6%) from migraine and tension-type headache, and 63 (33%) from nonclassifiable headache (for more details about clinical headache characteristics, see Table 1).

**Figure 2:**
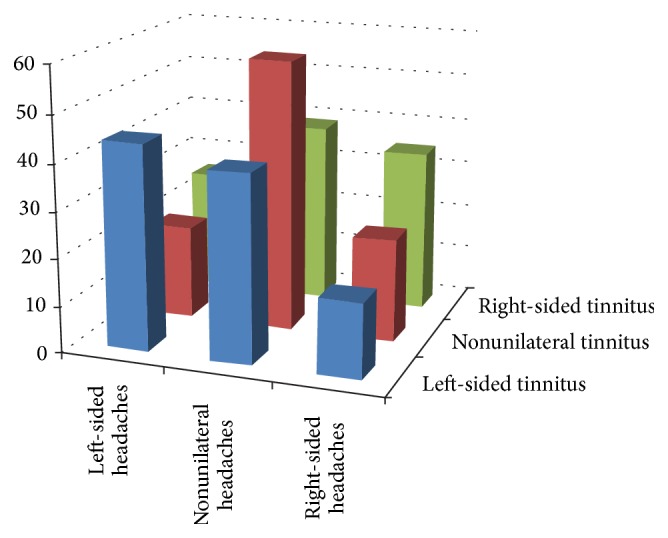
Prevalence rates of patients with headache laterality depending on tinnitus laterality (displayed as percent of all patients with a given tinnitus laterality). In all nonunilateral, left-sided, and right-sided tinnitus, the corresponding headache types were more frequently encountered.

There was a significant relationship between headache and tinnitus laterality (*χ* ^2^ = 15.490; df = 4; *p* = 0.004), even if among the headache types only the trigeminal autonomic headache is strictly side-locked. In all nonunilateral, left-sided, and right-sided tinnitus, the corresponding headache types were more frequently encountered (see Figure 2).

When asked about onset of tinnitus and headache, 67 (34.7%) patients reported tinnitus onset before headache onset, 106 (54.9%) patients reported headache onset before tinnitus onset, and 20 (10.4%) patients reported simultaneous onset. 101 (57.4%) patients reported that the onset of the second symptom did not influence the first; 60 (34.1%) patients reported worsening of the first symptom and in 15 (8.5%) the second symptom attenuated the first symptom. These three groups differed significantly in their TQ scores (*F* = 9.077; df = 2,162; *p* < 0.001) with the highest TQ scores in those patients in which the second symptom either increased (TQ: 50.93 ± 18.96; *p* < 0.001) or attenuated (TQ: 55.43 ± 13.12; *p* = 0.003) the first symptom, as compared to those without any change (TQ: 40.13 ± 17.65).

Asked about an ongoing relationship between tinnitus and headache, 82 (43.4%) reported that worsening of tinnitus was related to worsening of headaches and vice versa, and 9 (4.8%) patients reported an inverse relation (worsening of tinnitus related to improvement in headaches and vice versa). 79 (41.8%) reported no and 19 (10.1%) another relationship between tinnitus and headaches. Patients with an ongoing relationship between tinnitus and headache had higher TQ scores (positive relationship: 49.48 ± 16.76; inverse relationship: 45.44 ± 13.99; another relationship: 46.59 ± 19.04) than those without such a relationship (39.47 ± 19.06). ANOVA showed a significant main effect (*F* = 4.002; df = 3,173; *p* = 0.009) with a significant difference between the group with positive association and the group with no association (*p* = 0.001).

## 4. Discussion

In order to explore a potential relationship between tinnitus and headache we systematically investigated the occurrence of different headache types in a large sample of tinnitus patients. For this purpose, we used a self-report questionnaire, which has previously been used as a screening instrument in an epidemiological study in Germany [20] and in a study that analysed the relationship between headache and low-back pain [49]. In our sample of tinnitus patients, the proportion of tension-type headaches (19% of all headache patients) was clearly lower than the proportion of tension-type headache in the general population (44% of all headache patients [20]). The proportion of migraine patients in our sample was similar to that in the general population (50% of all headache patients in our sample as compared to 52% of all headache patients in the general population [20]) whereas unclassifiable headaches were slightly more frequent than in the general population (33% in our sample as compared to 26% in the general population [20]).

Our results demonstrate the feasibility to assess headache subtypes among tinnitus patients by using self-report questionnaires. However, we are also well aware about the shortcomings of the chosen approach. First, in the comparison of prevalence rates between our sample and the population-based survey, it has to be considered that the prevalence of the different headache forms depends strongly on age and gender, but the age and gender distribution of the tinnitus sample is not representative of the population. Second, we cannot exclude a selection bias and a reporting bias, since we only contacted tinnitus patients, who had presented at a tertiary tinnitus clinic and who had reported the existence of headaches, when they presented at the tinnitus clinic. Third, the retrospective design and the symptom assessment by questionnaires may be confounded by a recall bias. Fourth we received completed questionnaires only from about half of the patients, who had presented with tinnitus and headache in our clinic. The main reason for this relatively low response rate may have been that the interval between presentation in the clinic and contacting the patients was up to eight years.

Because of these limitations in a next step our findings should be complemented by case-control or population-based studies with prospective design and additional clinical examination to confirm prevalence rates of different headache forms among tinnitus patients. Also patient samples presenting in headache clinics should be investigated for the presence of tinnitus to obtain complementary information from patients in whom headache is the primary symptom. Face-to-face interviews will enable a higher diagnostic accuracy especially in patients with trigeminal autonomic cephalgias, in whom the validated questionnaire has only limited specificity [46, 48].

To our knowledge, our study is the first that studied the local and temporal relationship between headache and tinnitus in detail. Here we found a highly significant association between tinnitus laterality and headache laterality. An even higher correlation might be obtained by asking explicitly for side changes of headaches and tinnitus, which we did not do in this study. It may also be warranted to specifically screen for vestibular migraine, which may mimic Meniere's disease [42] and which can cause both tinnitus and headache.

With respect to symptom onset, more patients reported that headache onset preceded tinnitus rather than the opposite and only a small proportion reported that both symptoms started simultaneously. These findings were expected as headache typically starts at earlier age than tinnitus.

Thus, we observed a highly significant association between tinnitus and headache localisation and various possible temporal associations of the onset of the two symptoms. These data fit with the assumption that headache and tinnitus are linked via common pathophysiological mechanisms. As headache precedes tinnitus in the majority of cases one could assume that headache can trigger tinnitus. But tinnitus can also trigger headache or a third factor may predispose to local susceptibility (e.g., left, right, or nonunilateral). Thus, one could imagine that a unilateral headache syndrome results in sensitization of the trigeminal system, which then facilitates the development of tinnitus. Also the opposite direction (unilateral tinnitus sensitizing the trigeminal system and resulting in headache on the same side) is possible. Finally the susceptibility to both symptoms may be caused by a third factor, for example, a unilateral trigeminal pathology, a globally increased sensitivity for nonunilateral headache and tinnitus in the context of a somatization disorder, or an increased genetic susceptibility for developing thalamocortical dysrhythmia [29]. Further electrophysiological and neuroimaging studies will be needed to identify the neuronal link between both disorders.

About half of the asked patients reported that fluctuations in the symptoms are related to each other. In the vast majority of these cases worsening of one symptom went along with worsening of the other symptom, but there were also cases with a reciprocal interaction or a more complex relationship. Such a relationship of symptom severity over time is a further indicator for a pathophysiological link between tinnitus and headache. Patients reporting such a relationship had a significantly higher tinnitus severity as compared to patients where fluctuations of tinnitus and headache were not related. Since a relationship of the two symptoms over time can only be detected, if symptoms are fluctuating, the result is confounded by the existence of symptom fluctuations. Further studies are needed to distinguish whether fluctuations* per se* are related with higher symptom severity or whether it is the interrelation between headache and tinnitus that is responsible for higher tinnitus severity. It might be of particular interest to investigate those patients who reported a reciprocal interaction between tinnitus and headache, since the mechanisms of this interaction may provide hints for potential therapeutic interventions. While an increase of tinnitus during migraine attacks has been reported earlier [50, 51], a reciprocal interaction has not yet been described before.

In summary our findings of a significant relationship between tinnitus and headache laterality and a temporal interaction of both disorders in the majority of cases suggest that the cooccurrence of tinnitus and headache is not purely coincidental but that both disorders may be linked by shared pathophysiological mechanisms.

## Supplementary Material

Supplementary Material: Additional questions asked to the patients about the relationship between tinitus and headache

## Figures and Tables

**Table 1:** Prevalence and clinical characteristics of the different headache types (according to the questionnaire classification of Fritsche et al. (2007) [45]).

Headache type	*N* (% of whole sample)	Chronic (% of all patients with this headache form)	Episodic (% of all patients with this headache form)	Days with headache/month	Patients with medication intake (% of all patients with this headache form)	Days with medication intake/month
Migraine	86 (45%)	14 (16.3%)	72 (83.7%)	9.9 ± 9.3	66 (77%)	8.6 ± 11.9
Tension-type headache	25 (13%)	7 (28%)	18 (72%)	12.8 ± 8.7	16 (64%)	5.1 ± 3.5
Trigeminal autonomic headache	8 (4%)	1 (12.5%)	7 (87.5%)	8.5 ± 5.8	7 (87.5%)	13.7 ± 27.2
Migraine and tension-type headache	11 (6%)	Migraine: 2 (18.2%) Tension-type headache: 3 (27.3%)	Migraine: 9 (81.8%) Tension-type headache: 8 (72.7%)	13.1 ± 10.0	8 (73%)	7.6 ± 7.0
Nonclassifiable headache	63 (33%)	n.a.	n.a.	7.8 ± 6.9	43 (68.2%)	8.4 ± 8.6
