# Supplementary material for: Tinnitus and Headache
Source: Biomed Res Int. 2015 Oct 25;2015:797416. doi: 10.1155/2015/797416 (PMC4637068; doi:10.1155/2015/797416)
Supplement: Supplementary file 1 — Supplementary Material: Additional questions asked to the patients about the relationship between tinitus and headache [file 797416.f1.pdf]

Supplementary Material

Questions about headaches

25) On how many **days** per month are you perceiving **headaches**?

On about _________ days/month

26) Which **pain medication** are you taking because of your headaches?

Primary medication **(1):** _______________________________

Secondary medication **(2):** ______________________________

27) On **how many days per month** are you taking these medications?

medication **1** on about ____ days/month

medication **2** on about ____ days/month

Questions about the relationship between tinnitus and headache

28) Is your headache on one or predominantly one-sided ?

O Yes O No

If so, which side : O right O left

Is your tinnitus unilateral or predominantly one-sided ?

O Yes O No

If so, which side : O right O left

29) Did your headaches start earlier than the tinnitus

O Yes O No (If no, go to question 30)

In which year did headache and tinnitus start?

Onset of headaches : __________________

Onset of tinnitus : _________________

With the onset of tinnitus headaches O got worse O got better O remained unchanged

29) Did your tinnitus start earlier than your headaches

O Yes O No (If no, go to question 32)

In which year did tinnitus and headache start ?

Onset of tinnitus : _________________

Onset of headaches : __________________

With the onset of headaches tinnitus O got worse O got better O remained unchanged

31) Is there a relationship between headaches and tinnitus over time?

O when the tinnitus is stronger, the headaches are worse (and vice versa)

O when the tinnitus is stronger , the headaches are better (and vice versa)

O no relationship

O another relationship
